# Left Atrial Strain to Identify Diastolic Dysfunction in Children with Cardiomyopathies

**DOI:** 10.3390/jcm8081243

**Published:** 2019-08-17

**Authors:** Jolanda Sabatino, Giovanni Di Salvo, Costantina Prota, Valentina Bucciarelli, Manjit Josen, Josefa Paredes, Nunzia Borrelli, Domenico Sirico, Sanjay Prasad, Ciro Indolfi, Alain Fraisse, Piers E. F. Daubeney

**Affiliations:** 1 Department of Paediatric Cardiology, Royal Brompton Hospital, London SW36NP, UK; 2 Division of Cardiology, Department of Medical and Surgical Science, URT-CNR, Magna Graecia University, 88100 Catanzaro, Italy; 3 National Heart and Lung Institute, Imperial College, London SW36NP, UK

**Keywords:** atrial strain, diastolic function, cardiomyopathy, children

## Abstract

Background: Left ventricular (LV) diastolic dysfunction (DD) carries worse prognosis in childhood. 2-dimensional (2-D) left atrial (LA) strain accurately categorizes DD in adults but its role in children is unknown. Thus, the aim of this study is to investigate whether LA strain and strain rate could diagnose and classify DD in children with dilated (CMD), hypertrophic (HCM) and restrictive (RCM) cardiomyopathies (CM). Methods and Results: The study includes 136 children (aged 8.8 ± 6 years): 44 with DCM, 40 with HCM, 7 with RCM and 45 healthy controls (CTRL). They underwent standard echocardiographic examination and 2-D speckle-tracking analyses (LV longitudinal peak systolic strain (LS), LA peak systolic strain and strain rate). No significant differences in mitral E/A and pulmonary S/D ratios were observed among the four groups. Although E/E’ and indexed left atrial volumes were found to be significantly higher in HCM, DCM and RCM compared to CTRL (*p* < 0.001), they showed no significant difference among the three CM groups. LV LS values were significantly reduced in CM vs CTRL (*p* < 0.001) and in DCM vs HCM (*p* < 0.01), with no other differences between the remaining groups. LA peak systolic strain and strain rate values showed a steady and significant decrease with worsening of DD. Receiver Operating Characteristics (ROC) curves showed area under the curve of 0.976 (*p* < 0.001) for LA strain and 0.946 (*p* < 0.001) for LA strain rate, to distinguish CTRL from CMs. Conclusions: LA strain and strain rate could be a promising tool to better understand and classify DD in children with cardiomyopathies, opening the way to its clinical use.

## 1. Background

It is well known that left ventricular (LV) systolic function is associated with reduced outcomes in childhood cardiomyopathy (CM) [1,2]. Less is known about the role of LV diastolic dysfunction although some studies have suggested it may be associated with worse prognosis in childhood [3,4,5,6].

The evaluation of the LV diastolic function is still a challenge in paediatric patients. Although invasive atrial pressures and LV end-diastolic pressures are commonly employed as indices of ventricular stiffness and compliance, they are both influenced by other confounding factors [7,8]. Furthermore, CM paediatric patients are not routinely subjected to diagnostic cardiac catheterization.

Echocardiography still represents the cornerstone for the assessment and follow-up of diastolic dysfunction in children and young patients with CM. However, a recent study [8] demonstrated that diastolic parameters derived from adult studies are inadequate and not sufficiently discriminatory in childhood.

The left atrium (LA), via its reservoir, conduit, and booster functions, has a key role in modulating LV filling [9]. Many studies demonstrated the ability of 2-dimensional speckle tracking echocardiography to assess the atrial function also in children [10,11,12,13,14,15,16]. 

Recently, 2-D LA strain has been proposed to accurately categorize diastolic function in adults [17].

Unfortunately, there are very few data about LA strain in young patients with cardiomyopathies [18] and no data about its ability to study diastolic function in children and young patients.

Thus, the aim of our study is to investigate the utility of LA myocardial deformation properties to diagnose and classify diastolic dysfunction in children with hypertrophic (HCM), dilated (DCM) and restrictive (RCM) cardiomyopathies, in addition to standard echo Doppler criteria.

## 2. Methods

### 2.1. Study Population

The study population comprised 136 children (mean age 8.8 ± 6 years, Table 1), identified retrospectively from the institutional database of the Royal Brompton Hospital’s Paediatric Cardiomyopathy Service. The study was approved by the institutional ethics committee. 

Children were diagnosed with HCM (*n* = 40) if they had increased wall thickness in any myocardial segment (z score > 2.5) with normal/increased LV ejection fraction (EF) [8,19]. Measurements of LV wall thickness were performed from M-Mode acquisitions in short-axis views (mitral level, mid LV and apical level) at end-diastole. All LV segments were examined from base to apex. 

DCM (*n* = 44) was diagnosed in patients with LV EF < 50% and LV end-diastolic dimension z score > 2 [8,20] measured from M-Mode acquisitions; RCM was diagnosed in presence of impaired diastolic filling with preserved systolic function and normal ventricular-wall thickness [8,21].

All the echocardiographic variables were measured by the primary investigator. The echocardiogram used for the analysis was the first study performed at our Institution.

Moreover, 45 healthy controls (CTRL) were recruited from the Royal Brompton Hospital Outpatient Clinic. CTRL group encompasses subjects referred for atypical chest pain, otherwise healthy, not on any medication, and with normal cardiac evaluation, EKG, echocardiogram and performed stress echocardiogram (physical exercise).

### 2.2. Standard Echocardiography

Transthoracic echocardiographic studies were performed by using a commercially available ultrasound system (iE33 xMATRIX Philips Healthcare, Best, The Netherlands) equipped with adult, paediatric and neonatal transducers (S5-1, S8-3, S12-4), according to the standardized protocol of the Royal Brompton Paediatric Echolab. All data were transferred to a commercially available workstation (Xcelera, R3.2, Philips Healthcare, Best, The Netherlands) and analysed offline. Chamber size and function were assessed according to the latest guidelines [22]. Values for LV diameters and thickness were adjusted for body size and age expressed as z-scores according to the Boston Children’s Hospital z-score system [23]. 

All the patients underwent a comprehensive diastolic assessment. Diastolic standard parameters were measured by one expert paediatric cardiologist, according to the latest guidelines in adult patients [24]: mitral inflow early-to-late diastolic flow (E/A) ratio, mitral E wave deceleration time (DT), mitral lateral (E’lat) and septal (E’med) peak early diastolic tissue velocities, mitral E-to-mean E’ ratio, pulmonary venous (PV) systolic-to-diastolic peak velocity ratio (S/D), PV A wave reversal amplitude and duration, time difference between PV A wave reversal and mitral A duration. Finally, left atrial volume indexed to body surface area (LAVi) was derived from the apical four chamber view. Finally, the maximum tricuspid regurgitation velocity was used to estimate the peak tricuspid regurgitation gradient (PTG) by means of the modified Bernoulli equation. 

### 2.3. Two-Dimensional Speckle-Tracking Analysis

Two-dimensional speckle-tracking echocardiographic analyses were performed by a different experienced paediatric cardiologist (blinded to clinical and standard echo Doppler data), using TomTec 2-D (Cardiac Performance Analysis version 1.2.1.2).

For speckle tracking analysis, we acquired apical four-chamber views images using conventional two-dimensional gray scale echocardiography, during breath hold and with a stable ECG recording. To guarantee optimal tracking, three consecutive heart cycles were obtained at a frame rate of 50–80 frames/second, sinus rhythm and ≤10% variability in heart rate.

The LA endocardial border was traced in the apical 4-chamber view, excluding the appendage and pulmonary veins from the LA cavity. Then, LA longitudinal strain and strain rate curves were generated throughout the cardiac cycle. Accuracy of the automated border tracking was verified and manually adjusted if needed. The LA wall longitudinal lengthening, during the LV systole, was measured and used for the analysis (LA peak systolic strain and strain rate) [25]. 

Longitudinal strain (LS) of the LV was assessed using apical four-chamber view. The peak longitudinal strain values were calculated for each patient as previously described [26]. 

### 2.4. Cardiac Catheterization

Diagnostic cardiac catheterization was performed in 8 pts according to clinical indications. Of these, 2 had DCM, 1 HCM, 5 RCM. In only 1 child from the RCM group, cardiac catheterization was requested to be repeated a second time after four months. 

Cardiac catheterization was performed within 24 h after the echocardiographic study in 8 patients with clinical indication. LV pressure was obtained using a catheter-tipped micromanometer and recorded on a polygraph system, then the LV end-diastolic pressure was calculated.

### 2.5. Reproducibility Study

Interobserver variability for myocardial deformation properties was assessed in 15 randomly selected studies and was calculated as the ratio (expressed as a percentage) of the difference between the values obtained by each observer (expressed as an absolute value) divided by the mean of the two values and as intraclass correlation coefficients. Intraobserver variability was calculated by a similar approach. 

### 2.6. Statistical Analysis

Statistical analyses were performed using SPSS version 22.0 statistical package (SPSS Inc., Chicago, IL, USA). Distribution of variables was evaluated by visual inspection of frequency histograms and normality was tested using the Shapiro Wilk Test. Continuous variables were presented as mean (standard deviation). Comparisons among groups were performed using analysis of variance (ANOVA). The post hoc Bonferroni correction was used to account for multiple testing. 

The correlation of variables was calculated by linear regression analyses with determination of the Pearson’s correlation coefficient. 

The ability of atrial strain and strain rate values to discriminate diastolic dysfunction in children with CM was assessed using ROC Curve (Receiver Operating Characteristics Curve) with 95% confidence interval. We used the value of Area Under the Curve (AUC) = 1.0 to characterize the perfect discrimination [27]. In order to decrease the inflation of the Type 1 error rate due to multiple testing, the statistical significance was defined as two-sided *p* value < 0.01.

## 3. Results

Study population characteristics are described in Table 1. The overall population included 11 subjects <1 year of age, comprising two neonates, (age range 0–17 years). 

The HCM group had thicker ventricular septum and posterior walls, slightly smaller LV diameters and higher EF. The DCM group presented younger age than the other three groups, higher heart rate and lower LV EF. The RCM group was characterized by normal LVEF and significantly dilated atria (mean LAVi: 59 mL/m^2^ versus controls 17 mL/m^2^). 

Thirty-two DCM and five RCM patients were on medication for heart failure, including β-blockers (*n* = 26, 59% and *n* = 3, 43%, respectively), angiotensin-converting enzyme inhibitors (*n* = 32, 73% and *n* = 5, 71%, respectively), diuretics (*n* = 29, 66% and *n* = 3, 43%, respectively), and digoxin (*n* = 5, 11% and *n* = 0, 0%, respectively). Twenty-one HCM (52%) patients were on β-blockers.

### 3.1. Traditional Diastolic Parameters

**Figure 1:**
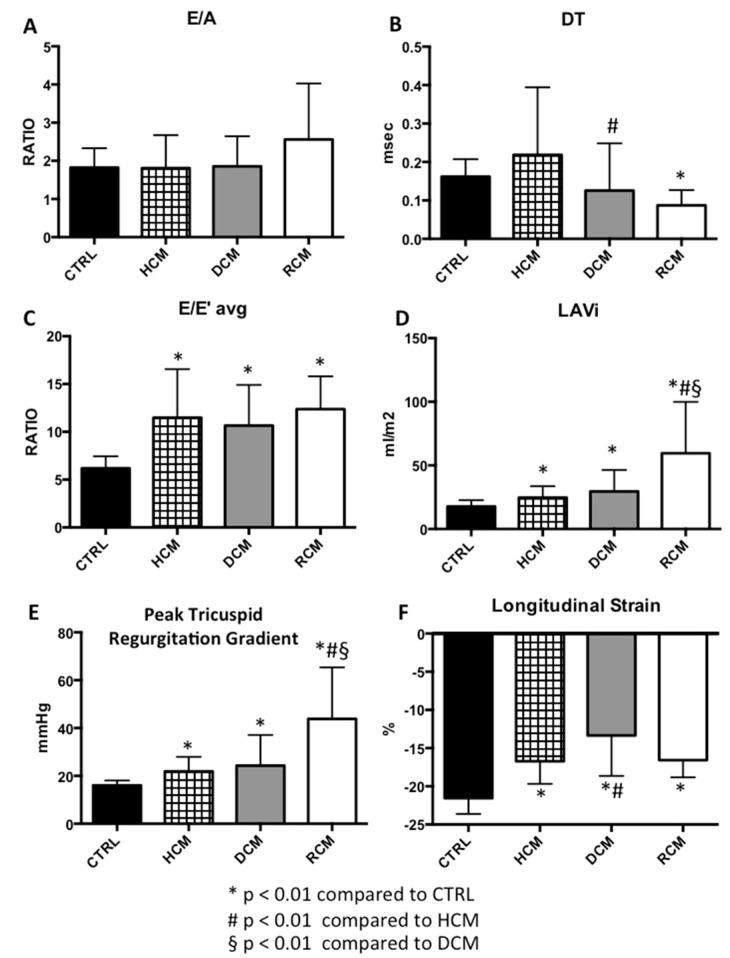
Comparisons of traditional diastolic echocardiographic parameters among three groups of paediatric cardiomyopathies and healthy controls. Mean and standard deviation of diastolic parameters in the study population by cardiomyopathy type. (**A**) E/A: mitral inflow peak E-to-A wave velocities ratio. (**B**) DT: mitral E wave deceleration time. (**C**) E/E’ avg: mitral inflow peak E-to-mean septal and lateral tissue velocities E’ ratio. (**D**) LAVi: left atrial volume indexed to body surface area. (**E**) PTRG: peak tricuspid regurgitation gradient. (**F**) LS: left ventricular longitudinal strain. * *p* < 0.01 compared to CTRL. ^#^ *p* < 0.01 compared to HCM. ^§^ = *p* < 0.01 compared to DCM. CTRL: normal controls. HCM: Hypertrophic Cardiomyopathy. DCM: Dilated Cardiomyopathy. RCM: Restrictive Cardiomyopathy.

The conventional diastolic parameters for each group are shown in Table 2. There were no significant differences in the mitral inflow E/A ratio among the HCM, DCM, RCM and the CTRL groups (Figure 1). 

MV DT significantly decreased in the RCM group (87 msec) compared to the CTRL (*p* < 0.001). DT was also significantly higher in the HCM group when compared to the DCM group (*p* = 0.006). No other significant differences in DT values were observed among the study groups (Figure 1).

The average E/E’ was found significantly higher in HCM, DCM and RCM compared to CTRL. There were no significant differences among the HCM, DCM and RCM groups (Figure 1).

LAVI was significantly larger in patients compared to CTRLs. Differences in LAVI were not significant between the HCM and DCM groups (Figure 1).

The peak systolic tricuspid pressure gradient was observed to be significantly higher in HCM and RCM compared to CTRL, and in RCM compared to DCM and HCM. No significant differences between HCM and DCM groups were noted (Figure 1).

The pulmonary S/D ratio showed no significant differences among the four groups.

### 3.2. Longitudinal Strain Analyses

Ventricular strain analyses were feasible in all the study samples. LV LS was significantly impaired in the cardiomyopathy patients compared to CTRL (*p* < 0.001). A significant impairment in LV LS was observed in the DCM group compared to the HCM group. No other significant differences in LV LS values were noted between the studied groups (Figure 1).

### 3.3. Atrial Strain Parameters

Atrial strain and strain rate analyses were feasible in all the study samples.

**Figure 2:**
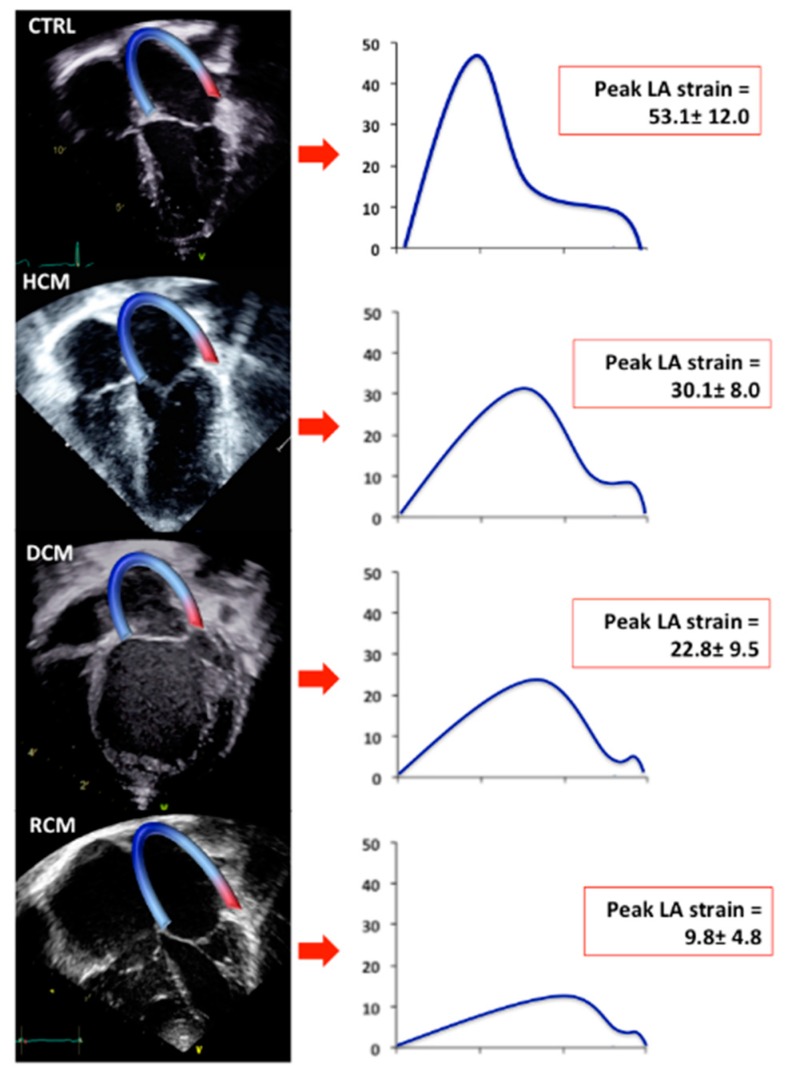
Speckle Tracking Analysis of Left Atrial Strain in the study population. Apical 4-chamber views of CTRL, HCM, DCM and RCM with the endocardium of the LA traced (**left**). On the (**right**), LA strain curves are depicted as mean of each studied group (blue curves). CTRL: normal controls. HCM: Hypertrophic Cardiomyopathy. DCM: Dilated Cardiomyopathy. RCM: Restrictive Cardiomyopathy. LA: Left Atrial.

**Figure 3:**
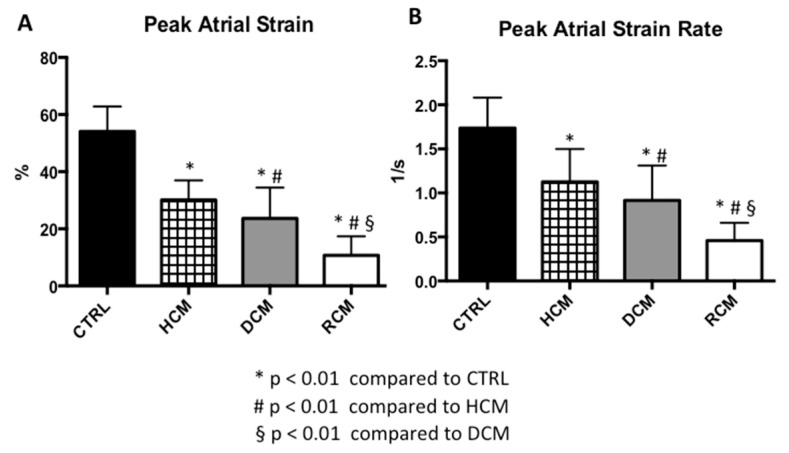
Comparisons of left atrial strain and strain rate values among three groups of paediatric cardiomyopathies and healthy controls. Mean and standard deviation of peak left atrial strain (**A**) and strain rate (**B**) in the study population by cardiomyopathy type. * *p* < 0.01 compared to CTRL. ^#^ *p* < 0.01 compared to HCM. ^§^ = *p* < 0.01 compared to DCM. CTRL: normal controls. HCM: Hypertrophic Cardiomyopathy. DCM: Dilated Cardiomyopathy. RCM: Restrictive Cardiomyopathy.

Peak systolic LA strain and strain rate values demonstrated a significant progressive decrease along the four groups (Table 2, Figure 2 and Figure 3). 

No significant correlations were found between peak systolic LA strain and the E/A ratio (r = −0.118, *p* = 0.193). Significant and moderate correlations were found between peak systolic LA strain or strain rate and the average E/E’ (r = 0.492, *p* < 0.001 and r = 0.513, *p* < 0.001, respectively). Also, a moderate correlation was found between LA strain and peak tricuspid regurgitation gradient (r = −0.571, *p* < 0.001), and between LA strain and LAVI (r = −0.490, *p* < 0.001).

**Figure 4:**
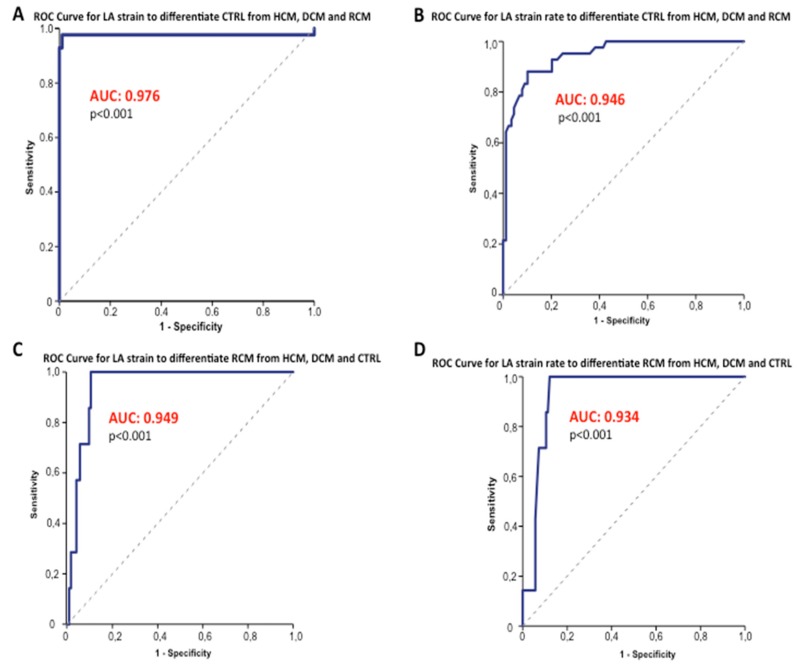
LA strain and strain rate Receiver-Operating Characteristic Curves. The ROC Curve showed optimal capability for LA strain and strain rate (**A**,**B**) to discriminate healthy children with normal diastolic function from patients with paediatric cardiomyopathies (LA strain AUC = 0.976, *p* < 0.001; LA strain rate AUC = 0.946, *p* < 0.001). Panel (**C**,**D**) shows ROC Curves (LA strain AUC = 0.949, *p* < 0.001; LA strain rate AUC = 0.934, *p* < 0.001) which differentiate RCM from the other three study groups (HCM, DCM and CTRL). ROC = receiver-operating characteristic. AUC: area under the curve. CTRL: normal controls. HCM: Hypertrophic Cardiomyopathy. DCM: Dilated Cardiomyopathy. RCM: Restrictive Cardiomyopathy. LA: Left Atrial.

**Figure 5:**
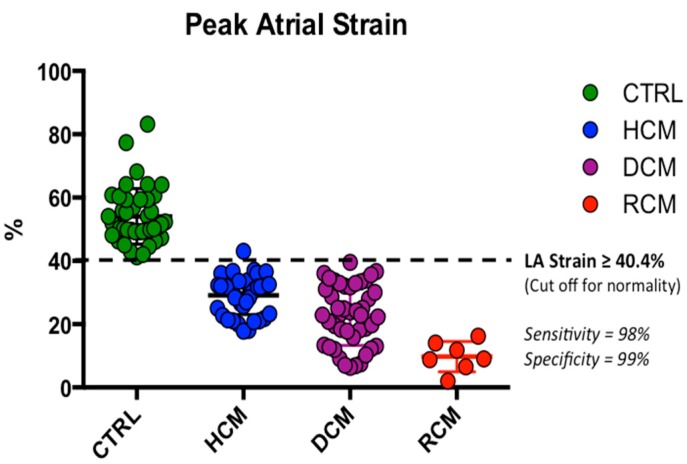
LA Strain cut-off to differentiate normal diastolic function in paediatric age. The dotted line represents the cut-off (≥40.4%; sensitivity, 98%; specificity, 99%) obtained to differentiate children with normal diastolic function (green dots), from those with HCM (blue dots), DCM (purple dots), RCM (red dots). CTRL: normal controls. HCM: Hypertrophic Cardiomyopathy. DCM: Dilated Cardiomyopathy. RCM: Restrictive Cardiomyopathy. LA: Left Atrial.

Receiver-operating characteristic curves showed area under the curve (AUC) values of 0.976 (*p* < 0.001) and 0.946 (*p* < 0.001) for LA peak systolic strain and LA peak systolic strain rate to differentiate normal young individuals from the three groups of juvenile cardiomyopathies (LA strain cut-off value ≥ 40.4%; sensitivity, 98%; specificity, 99%; LA strain rate cut-off value ≥ 1.39 1/s; sensitivity, 88%; specificity, 90%) (Figure 4 and Figure 5). 

The studied LA myocardial deformation properties demonstrated excellent ability to discriminate RCM from HCM, DCM and control subjects (LA strain AUC = 0.949, *p* < 0.001; LA strain rate AUC = 0.934, *p* < 0.001) (Figure 4). 

### 3.4. Left Atrial Strain Correlation with Invasive End-Diastolic Pressures

We were able to collect a total of nine invasive LV end-diastolic pressures from eight children (mean age 9 ± 7 years). 

**Figure 6:**
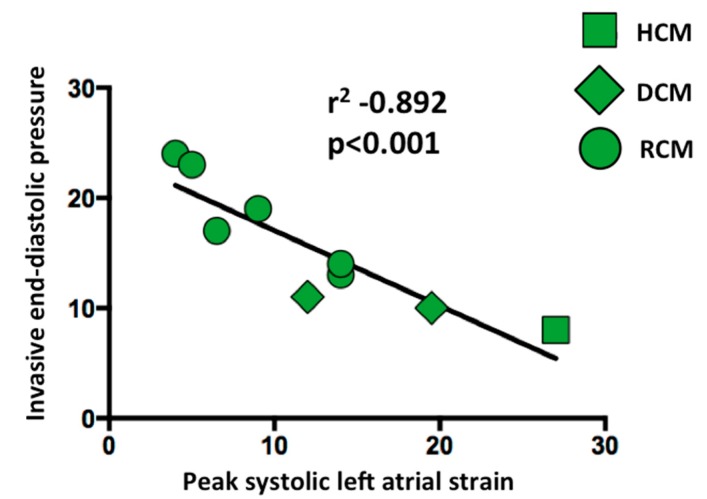
Linear correlation between left ventricular end-diastolic pressures and left atrial peak systolic strain. The graph shows a strong significant inverse correlation between peak systolic LA strains and invasive LV end-diastolic pressures (r = −0.892, *p* < 0.001). HCM: Hypertrophic Cardiomyopathy. DCM: Dilated Cardiomyopathy. RCM: Restrictive Cardiomyopathy.

Univariate regression analysis demonstrated that peak LA strain had a strong significant inverse correlation with invasive LV end-diastolic pressure (r = −0.892, *p* < 0.001) (Figure 6). On the other hand, invasive LV end-diastolic pressure had non significant correlations with E’ avg (r = −0.139, *p* = 0.721), E/E’ avg (r = 0.238, *p* = 0.537), MV DT (r = 0.485, *p* = 0.186) and LAVi (r = 0.514, *p* = 0.157).

### 3.5. Reproducibility Analyses

Intra- and inter-observer variability expressed as the mean percentage error (absolute difference/mean) and the intraclass correlation coefficients were optimal for LV longitudinal strain (7 ± 7% and 7 ± 8%, respectively; intraclass correlation coefficients: 0.92 and 0.93, respectively) and for LA peak systolic strain (14 ± 4% and 15 ± 6%, respectively; intraclass correlation coefficients: 0.89 and 0.87, respectively), good for LA peak systolic strain rate (14 ± 3% and 15 ± 3%, respectively; intraclass correlation coefficients: 0.88 and 0.87, respectively).

## 4. Discussion

The results of this study demonstrate, for the first time, that the assessment of LA strain and strain rate, using 2-D speckle tracking analysis, may be an accurate diagnostic tool for functional evaluation of different cardiomyopathies in children and young patients, with a strong significant correlation with invasive LV end-diastolic pressure. These findings represent a positive change in evidence, because standard echo-Doppler criteria can classify diastolic dysfunction only in a small proportion of CM young patients, even when their disease is severe [8]. 

Our findings are particularly relevant because of the lack of the availability of reference values for diastolic dysfunction in childhood. Indeed, the sole existing guidelines and recommendations are currently derived from adult studies [24]. 

### 4.1. Left Atrial Strain and Standard Echo Doppler

In this study, although we analysed a number of traditional echocardiographic and Doppler parameters [24], we observed that only the peak systolic LA strain and strain rate changed progressively and remained significantly different through all the spectrum of functional abnormalities observed in the paediatric and young HCMs, DCMs and RCMs. Furthermore, in our studied cohort, the LA strain and strain rate cut-offs (LA strain cut-off value ≥ 40.4%; sensitivity, 98%; specificity, 99%; LA strain rate cut-off value ≥ 1.39 1/s; sensitivity, 88%; specificity, 90%) were able to categorize young patients with normal diastolic function. 

Dragulescu et al. suggested that E’, DT and LAVi appeared to be the most helpful, among the other conventional echo parameters, in the evaluation of diastolic function in pediatric CM (175 patients age range 0–18) [8]. Nevertheless, they found these parameters allowed diagnosis of diastolic dysfunction only in a small proportion of patients and they found these echo parameters not sufficiently discriminatory preventing further classification. In this context, we observed a good correlation between either LA strain or strain rate and LAVi (r = 0.730). However in our study, we observed no significant difference between children with HCM and DCM in LAVi values. 

Moreover, the mean DT, compared to the one observed in healthy children, significantly decreased in RCM, but lost significance between the more severe grades of diastolic dysfunction, i.e., DCM and RCM. 

In our study, E/E’ was only able to differentiate normal young subjects from those with CM, without the ability to further discriminate through the various CMs in young patients.

Of interest, in the subgroup of patients who underwent cardiac catheterization no significant correlation was found between LAVi, E/E’ avg, E’ avg, MV DT and LV end-diastolic pressure.

By comparison, LA strain and strain values were able to identify diastolic dysfunction with high accuracy (LA strain cut-off value ≥ 40.4%; sensitivity, 98%; specificity, 99%). In addition, atrial strain myocardial deformation properties were significantly different among all the CM groups, differentiating RCM from all the other cardiomyopathy groups. These findings suggest that LA myocardial deformation properties may be a key indicator of LA dysfunction that occurs with the gradual progression of diastolic dysfunction. 

Our findings are in agreement with Singh A et al. who elegantly demonstrated that the LA strain can categorize the LV diastolic dysfunction in adult patients, because unlike the traditional parameters, it changes progressively and significantly through all the grades and the severity of the diastolic dysfunction [17]. 

In this study we observed a significant and moderate correlation between LA strain and peak tricuspid regurgitation gradient (r = −0.571, *p* < 0.001). However, although we found that peak systolic tricuspid regurgitation gradient was significantly higher in HCM and RCM compared to CTRL, there were no significant differences among all the study groups. 

These findings are in agreement with previous papers suggesting that elevated levels of pulmonary arterial systolic pressure are closely related to elevated LV filling pressures and/or enlargement of LA volumes in adult patients [24,28]. 

### 4.2. Left Atrial Strain and Progressive Diastolic Dysfunction

It is well known that the LA behavior consists of modulating the LV filling through its function of reservoir, conduit and booster pump. The reciprocal relationship existing between the distributions of LA functions is a sort of compensatory mechanism that facilitates the LV filling in patients with myocardial disease [29]. 

Atrial reservoir phase is fundamental for LV filling, by storing energy during ventricular systole that is released after the mitral valve opening. 

It has been demonstrated in adults that peak systolic LA strain has a strong inverse correlation with invasively assessed LV filling pressures [30,31]. In line with these findings, we demonstrated, for the first time in children, a strong inverse correlation between peak systolic LA strain values and the LV end-diastolic pressures measured invasively in nine studies. The potential mechanism of such inverse correlation could be explained by the assumption that the left ventricular end diastolic pressure acts as the afterload of the LA function; indeed, if the left ventricular end diastolic pressure is high, the LA is subject to mechanical stresses which progressively reduces the LA reservoir function [30].

### 4.3. Study Limitations

This is a single-centre study. The DCM study group encompasses a relatively younger population compared to the other two groups of CM; it is also difficult to determine whether patients of the same group were at the same time point in the disease process. However, this reflects the variety encountered in the real clinical practice. Similarly, since the duration of different heart failure therapies may have an impact on the assessment of diastolic function, we used in our study the first echocardiogram performed at our Institution. It was considered not ethical to stop cardiac medication for the assessment of diastolic function.

We could not correlate atrial strain values with invasive LV filling pressures in the whole population, since these were not available, retrospectively, and cardiac catheterization is not routinely performed in these patients. However, we were the first to study the relation between LA strain and LV end-diastolic pressure in children. In addition, we demonstrated that atrial strain and strain rate can accurately stratify diastolic dysfunction as opposed to standard echo Doppler parameters. 

Finally, the diagnostic accuracy of the cut-off values of LA strain and strain rate proposed in our study should be confirmed in larger prospective studies.

## 5. Conclusions

Our findings suggest that LA myocardial deformation analysis, by 2-D speckle tracking, is feasible and could be a promising tool to better understand and classify diastolic dysfunction in children and adolescent patients, while conventional echo-Doppler parameters have only a limited ability. Our findings, if confirmed by other studies, may open the way to a novel diagnostic approach for the assessment of diastolic function in the paediatric age range.

## Figures and Tables

**Table 1:** General characteristics of the studied cohort.

	Groups			
General and Echocardiographic Variables	CTRL (N = 45)	HCM (N = 40)	DCM (N = 44)	RCM (N = 7)
Age (years)	10.4 (4.7)	10.9 (5.5)	5.0 (5.2)	10.0 (7.0)
Male, n (%)	18 (40)	24 (61)	21 (48)	3 (43)
Body Surface Area (BSA) (m^2^)	1.2 (0.5)	1.3 (0.5)	0.8 (0.5)	1.1 (0.6)
Mean Heart Rate (bpm)	85 (15)	71 (22)	115 (18)	95 (18)
LVEDD (mm)	39.6 (7)	34.5 (8) *	44.5 (10) ^#^	35.3 (9)
LVEDD Z score	−0.5 (0.8)	−1.7 (0.5) *	3.5 (0.2) *^,#^	−1.1 (0.4) ^§^
IVS (mm)	6.1 (1)	13.5 (5) *	5.1 (2) *^,#^	7.1 (2) ^#^
IVS Z score	−0.4 (0.7)	3.1 (0.2) *	-0.9 (0.9) ^#^	1.2 (0.7) ^§^
LVEF (%)	60 (4)	68 (7) *	39 (12) *^,#^	59 (8) ^#,§^

Values are mean (SD), or *n* (%). * *p* < 0.01 compared to CTRL. ^#^ *p* < 0.01 compared to HCM. ^§^ = *p* < 0.01 compared to DCM. LVEDD: Left Ventricular End Diastolic Diameter. IVS: Interventricular Septum. LVEF: Left Ventricular Ejection Fraction. BSA: Body Surface Area. CTRL: normal controls. HCM: Hypertrophic Cardiomyopathy. DCM: Dilated Cardiomyopathy. RCM: Restrictive Cardiomyopathy.

**Table 2:** Studied standard echo-doppler parameters and strain values.

	Groups			
Diastolic Parameters	CTRL (N = 45)	HCM (N = 40)	DCM (N = 44)	RCM (N = 7)
MV E vel (cm/sec)	90.2 (13.8)	95.6 (19.9)	98.4 (25.8)	80.0 (33.0)
MV A vel (cm/sec)	53.2 (14.7)	59.2 (20.3)	58.0 (20.1)	34.8 (20.3)
MV E/A (ratio)	1.8 (0.5)	1.8 (0.9)	1.8 (0.8)	2.5 (1.5)
MV DT (msec)	162 (45.8)	219 (175.7)	125 (123) ^#^	87 (40) *
MV E’ sep (cm/sec)	13.6 (2.0)	8.3 (3.0) *	9.3 (2.8) *	6.2 (1.8) *^,§^
MV E’ lat (cm/sec)	16.8 (3.4)	11.2 (4.1) *	11.3 (4.1) *	8.0 (4.1) *
MV E/E’ avg	6.2 (1.2)	11.5 (5.5) *	10.7 (4.2) *	12.4 (3.4) *
LAVi (mL/m^2^)	17.7 (4.9)	24.6 (9.0) *	29.6 (16.8) *	59.6 (40.3) *^,#,§^
PTRG (mmHg)	16.1 (2.0)	21.9 (6.1) *	24.3 (12.8) *	43.8 (21.5) *^,#,§^
PV S/D	1.0 (0.3)	1.3 (0.3)	1.0 (0.3)	1.4 (0.7)
**Atrial and ventricular strain parameters**				
LV LS (%)	−21.5 (2.1)	−16.7 (3.0) *	−13.3 (5.3) *^,#^	−16.6 (2.3) *
LA peak systolic strain (%)	53.1 (12.0)	30.1 (8.0) *	22.8 (9.5) *^,#^	9.8 (4.8) *^,#,§^
LA peak systolic strain rate (1/s)	1.7 (0.3)	1.1 (0.3) *	0.9 (0.4) *^,#^	0.4 (0.2) *^,#,§^

Values are mean (SD), or n (%). * *p* < 0.01 compared to CTRL. ^#^ *p* < 0.01 compared to HCM. ^§^ = *p* < 0.01 compared to DCM. CTRL: normal controls. HCM: Hypertrophic Cardiomyopathy. DCM: Dilated Cardiomyopathy. RCM: Restrictive Cardiomyopathy. MV E vel: mitral inflow peak E velocity. MV A vel: mitral inflow peak A velocity. MV E/A: mitral inflow peak E-to-A wave velocities ratio. MV DT: mitral E wave deceleration time. MV E’ sep: peak early diastolic tissue velocity at medial mitral annulus. MV E’ lat: peak early diastolic tissue velocity at lateral mitral annulus; MV E/E’ avg: mitral inflow peak E-to-mean septal and lateral tissue velocities E’ ratio. LAVi: left atrial volume indexed to body surface area; PTRG: peak tricuspid regurgitation gradient. PV S/D: pulmonary venous peak systolic-to-diastolic velocity ratio; LV LS: left ventricular longitudinal strain. LA strain: left atrial peak strain. LA strain rate: left atrial peak strain rate.
